# Adapting Medical Education Initiatives Through Team-Based e-Learning, Telemedicine Objective Structured Clinical Exams, and Student-Led Community Outreach During the COVID-19 Pandemic

**DOI:** 10.2196/26797

**Published:** 2021-06-14

**Authors:** Julia H Miao

**Affiliations:** 1 Department of Biological Sciences Cornell University Ithaca, NY United States; 2 Department of Medicine Renaissance School of Medicine at Stony Brook University Stony Brook, NY United States

**Keywords:** medical education, COVID-19, medical student, community service, telemedicine, telehealth, community outreach, peer teaching, student-led initiative, clinical assessment, adaptability, team-based learning

## Abstract

Although the COVID-19 pandemic has quickly prompted medical schools and students around the world to transition from their traditional classrooms to web-based learning, the global crisis has inspired the development of innovative e-learning solutions that use existing technology and other web-based tools to continue nurturing the education of medical students while ensuring the public health and safety of both students and faculty members alike. Through the perspective of medical students, we share how the COVID-19 pandemic has impacted and transformed small team–based learning in medical education; changed objective structured clinical exam evaluations and the practice of clinical skills through telemedicine; and nurtured nationwide, web-based, student-led initiatives for community outreach, telehealth, and medical services.

## Introduction

In today’s day and age of innovative technology, the urgency and necessity of social distancing during the COVID-19 pandemic has paved the way for the rapid reimagination and flexible restructuring of medical school education. As medical students across the country trickled out from their traditional lecture halls and classrooms to transition to web-based learning, the global crisis prompted the development of innovative e-learning solutions. These solutions used existing technology and other web-based tools to continue nurturing the education of medical students while ensuring the public health and safety of both students and faculty members alike. In this paper, we highlight the challenges that arose during the pandemic and the solutions that were embraced to address them. These solutions served as important methods that were implemented on web-based platforms during the pandemic for medical students and faculty members in medical school curricula, and they will play essential roles in medical education during and after the pandemic in the future. There is no doubt that in-person medical education and face-to-face clinical interactions are invaluable to medical students’ learning. Although the pandemic has disrupted traditional, in-person medical education, new pedagogical approaches that integrate technology and embrace flexibility among students and professors have helped to empower and connect students during a time of social distancing. The transition to web-based platforms does not replace traditional, in-person medical education but rather transforms, adapts, and enriches the best learning practices during these challenging pandemic times. Through the perspective of medical students, we share how the COVID-19 pandemic impacted and transformed small team–based learning in medical education; changed objective structured clinical exam (OSCE) evaluations and the practice of clinical skills through telemedicine; and nurtured nationwide, web-based, student-led initiatives for community outreach, telehealth, and medical services. These insights highlight how students have continued to engage in collaborative learning and increasing their resilience in medical school during times of crisis. They also offer new, enriching perspectives on maximizing the uses of advancing technology for organizing and delivering effective medical content.

## Challenges and Solutions: Small Team–Based e-Learning and Teaching During the COVID-19 Pandemic

Many recent research articles focus on the transition from medical school lectures in the classroom to web-based learning [1,2]. Certainly, the transition to web-based medical school education has its own challenges and solutions. The advent of more flexible technologies, including Zoom (Zoom Technologies Incorporated) and Microsoft Teams recordings, that not only offer live lectures for students but also supplement recordings with annotated captions and helpful time stamps for students anywhere and anytime allow students to return to, rewind, and replay lectures at their own individual paces. These technology functionalities as well as other features, such as real-time global cloud quiz polling and breakout rooms for small-group learning, have helped ease the transition to web-based medical school education [3]. In this section, we provide a unique perspective on small team–based learning in medical school education during the COVID-19 pandemic.

The pandemic has raised important questions about how we can reconcile the loss of small, in-person, team-based learning, such as working together on patient cases or discussing medical topics as a group of students. Here, we highlight adaptable technologies that have assisted in medical education through small group–based learning. One of the key functions of Zoom is breakout rooms, wherein students are preassigned to small groups or randomly assigned to one of these smaller rooms with other randomly assigned students to discuss a health care–related topic or complete an assignment. After students conduct discussions together in their mini breakout rooms like a small team, the rooms reconvene with the larger lecture hall or the main Zoom screen, with all participants shown in gallery view, to provide their insights to the whole class via the elected team student representative.

The adaptive uses of these technologies demonstrate that small team–based learning in web-based medical schools can also be facilitated; nevertheless, the feasibility of using breakout rooms to facilitate small team–based learning did not come without its initial challenges. When it came to large classes of over 100 students requiring assignment to certain breakout rooms, faculty professors often needed several minutes in early sessions to preassign students to individual groups of ≤8 people. This function was not feasible for Zoom features, as only the assigned hosts were able to designate certain students to certain breakout rooms. Of course, random assignment to breakout rooms was feasible with an easy click of the button, but the preassigned grouping of large amounts of students proved to be more challenging. Alternate video-based classrooms with breakout rooms include Cisco Webex and Google Meet, which work similarly to Zoom. The Cisco Webex breakout room feature allows students to assign themselves individually to their designated breakout room without needing a single host or professor to assign hundreds of eager students. Although this has its advantages, students can easily enter other rooms at any time, leading to potential chaos, especially during preassigned group medical quizzes. Google Meet also has a web feature for breakout rooms yet only provides the moderator or the professor with the ability to control who gets assigned to which breakout room. Nonetheless, the successes of breakout room technology will help us to continue to integrate and adapt small team–based learning and teaching; they will also help with identifying areas for technology improvement that can further enhance and assist medical education applications in the near future.

Other web-based tools that medical students found incredibly helpful during preclinical, team-based learning were Google Docs and Microsoft Teams’ Word, which allow students to simultaneously contribute to, add, and revise team assignments. Comments can be added easily throughout a page; they pop up as bubbles in the margin column. In this column, classmates and teammates can teach each other and learn together by addressing each other’s questions or comments in order to complete assignments or reports on medical cases.

Web-based, peer-to-peer teaching and e-learning during the COVID-19 pandemic have also resulted in a collegial environment between classes that was built through web-based medical education [4]. For preclinical students, web-based social events that integrate peer-to-peer teaching and medical education included Medical Jeopardy and therapeutic art classes that nurtured creative cognition and enhanced visual observation skills that are integral to performing clinical diagnostics and addressing patients’ health. Medical student–led organizations and interest groups also showcased medical movie documentaries through Zoom sessions and led discussions. These were both informative and fun and provided a unique medical perspective on various social determinants of health. These socially distanced yet social events not only helped to unite medical classmates in a web-based and fun e-learning environment but also helped to foster interpersonal connections and offered places to destress and learn together.

## Challenges and Solutions: Adapting Medical OSCEs Through Telemedicine

Obtaining clinical skills through practice and assessment is integral to building the clinical foundations of medical students, who are often assessed through OSCEs. These learning opportunities, in which students take a patient history, perform a physical exam, create a differential diagnosis, and counsel standardized patients, are often video recorded and replayed by students to evaluate and improve clinical, lifelong learning skills. OSCEs are formal, objective evaluations that are continuously conducted throughout medical school training to develop and assess the clinical learning and skills of medical students. During the pandemic, these skills were put to the test through a flexible and web-based adaptation of prepandemic OSCE patient encounters via telemedicine and telehealth OSCEs [5,6].

In the beginning of the OSCE, to ensure patient privacy and confidentiality before beginning each patient encounter, we medical students assessed for an appropriate video background on Zoom in order to ensure that no person (or pet) enters the room and interrupts the session, much like they do for a physical patient care room where physicians and patients interact. The confirmation of medical student identity by physically showing our IDs to the camera and the confirmation of patient identity on web-based platforms are essential.

During the interactions, evaluations in telemedicine OSCEs ensured that we maintained adequate eye contact and established human connection and rapport, just as we would in a regular in-person OSCE. For many of us medical students, the web-based OSCE, which was conducted during the COVID-19 pandemic, was one of our first encounters with telemedicine. Balancing note-taking and eye contact via the new technology functionalities at first was tricky, but with practice, we were able to navigate potential technological glitches while flexibly managing volume controls and visibility. For example, in one of our OSCEs, we interviewed a geriatric patient who was hard of hearing and his family member and learned to balance eye contact with both participants on separate screens while also maintaining patience and empathy through patient-centered care and medical counseling. We have continued to recognize the limitations of meeting patients on a web-based platform and miss the physical handshakes and compassionate touches that occur during a physical exam. Thus, we compensated and practiced appropriate facial expressions, strong verbal communication, and maintaining our awareness of body language through a web-based medium. The telehealth OSCE provides us with the opportunity to continue to create a cohesive, fluid conversation between medical students and patients and further strengthen the bond of a physician-patient relationship through clinical practice and reflection [7].

Telemedicine is likely to be a vital part of future patient care. Physical exams and the development of hands-on skills during the pandemic and distance learning for medical students were certainly a challenge. Medical schools have stepped up and created safe learning environments for the development of physical exam skills that allow for social distancing. Students learn and practice physical exam skills during the nonclinical years of school prior to in-person clerkships on mannequins, standardized patients, and student pairs with full personal protective equipment (PPE), including masks. Simulating the pandemic world with social distancing and OSCEs played a vital role in the development of physical exam skills for both medical students and patients.

As the number of patient cases of COVID-19 began to rise nationally during the fall of 2020, we continued to implement OSCEs through telemedicine. During a web-based clinical medicine bootcamp, we medical students worked in pairs and observed our partners interviewing a patient while obtaining a focused medical history, recording chief complaints, and providing differential diagnoses. Feedback from student pairs became essential for continued improvement and learning. Furthermore, we concluded each patient exam with a write-up of a patient note, during which fourth year medical studies helped with mentoring the preclinical second-year students and provided them with OSCE-related advice.

As always, just like with any other OSCE, demonstrating the qualities of genuine concern, compassion, respect, and support validated our patients’ feelings and perspectives through empathy. Developing these important soft skills while also practicing objective clinical skills gained from medical content and knowledge is critical for the development of a physician in training [8]. These valuable learning opportunities have become an alternative to cancelled in-person OSCEs during the pandemic and have helped provide medical students and health professional trainees with an insightful clinical experience while telehealth rises in critical importance [9,10]. We obtained real-time feedback from supportive classmates in a supervised environment as well as feedback from both upperclassmen and faculty mentors. These live, interactive scenarios; the practice of medical counseling; and medical history taking have enhanced medical education.

## Challenges and Solutions: Web-Based, Student-Led Initiatives in Community Outreach and Patient Care During the COVID-19 Pandemic

Sidelined from the front lines of the pandemic but passionate about contributing to the efforts in any way they can, medical students across the nation have helped initiate and have engaged in patient care–related community service initiatives during the COVID-19 pandemic. Such services have included engaging in video calls with nursing home residents to keep them company and to keep them connected during isolation periods and volunteering for the discharge counseling of recovering patients with COVID-19 via telehealth [11]. These community service projects, in conjunction with medical education, have become a vital part of our growth as medical students and clinical service learning during the COVID-19 pandemic.

Through a symphony of teamwork, medical students with diverse backgrounds, interests, and skills channel their energy to integrate their passions to elevate local and global community health in any way they can. Gathered together, medical students mirror an orchestra as various communities with their unique individual skills come together to collaborate, share, aid those in need [12]. Many medical student–led initiatives have included medical outreach for those who are underserved and socioeconomically disadvantaged [13-15]. For example, one student-led initiative involved weekly check-ups, phone calls, or video calls with older adults at local, underserved nursing homes to not only provide social support during their isolation but also enhance their physical and mental health through medical counseling [14]. Other groups of collaborating medical students from multiple medical institutions nationwide have fostered teamwork to create a contactless service and apply their multidisciplinary skills in linguistics, graphic design, and verbal and written communication [11,15]. They created and translated public health pamphlets in multiple languages for diverse community members across the country. Other students who were passionate about technology and hands-on projects initiated the 3D printing of face masks to address the nationwide shortage of PPE and masks and provide PPE to both health care workers and communities [11]. They used technology to help with the 3D printing and physical assembly of face masks for frontline health care workers. Although these student-led initiatives and extracurriculars may not directly stem from a medical school’s standard curriculum, these student initiatives leverage web-based resources and are vital to medical students’ education as they serve their communities and deliver compassion.

## Conclusion

Despite social distancing and web-based challenges, both medical students and medical education faculty members have stepped up and risen to the challenge as they learn and grow together to navigate a digitally transformed curriculum during the COVID-19 pandemic. Technology has helped to pave the way for flexibility in medical education via web-based adaptation during this global pandemic. Such technologies have also provided medical students with the necessary resources for continual learning in their medical education, social connection, and service to their greater communities. There is no doubt that this pandemic has created a challenging time for many people, including medical students, medical professors and faculty members, families, friends, loved ones, and communities. Ranging from the various implemented technologies such as small, web-based breakout rooms to technologies that allow for the adaptable integration of telemedicine into OSCEs and clinical practice, e-learning tools have helped us to navigate these challenges together with resilience and flexibility. These technical innovations and learning chances are valuable and should be continued during and after the pandemic because they minimize travel time and increase the flexibility of learning, thereby allowing learning to occur anywhere and anytime among medical students and faculty members. For example, the advent of these technological tools, which have been integrated into our web-based medical education, also helped pave the way for innovative modalities in medical education. These modalities include web-based, team-based learning and the enhanced practice of telemedicine with patients, which has increasingly grown in importance during the 21st century and times such as the COVID-19 pandemic. With optimism and hope, we—strengthened in unity and resilience—look forward to continuing to embrace the innovative initiatives of our current web-based medical education as future physicians of tomorrow.

## Abbreviations

OSCE: objective structured clinical exam
PPE: personal protective equipment
